# Supplementary figures and images for: Vascular Growth Factor Inhibition with Bevacizumab Improves Cardiac Electrical Alterations and Fibrosis in Experimental Acute Chagas Disease
Source: Biology (Basel). 2023 Nov 10;12(11):1414. doi: 10.3390/biology12111414 (PMC10669550; doi:10.3390/biology12111414)

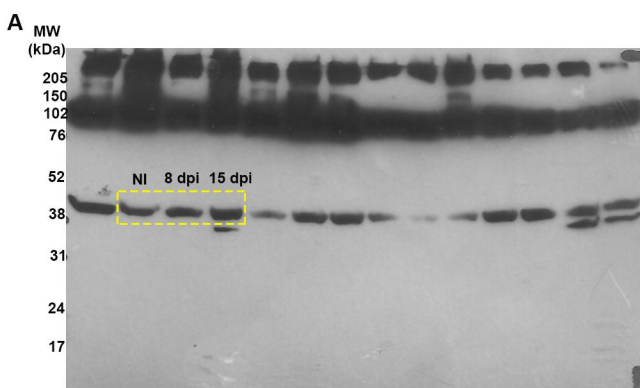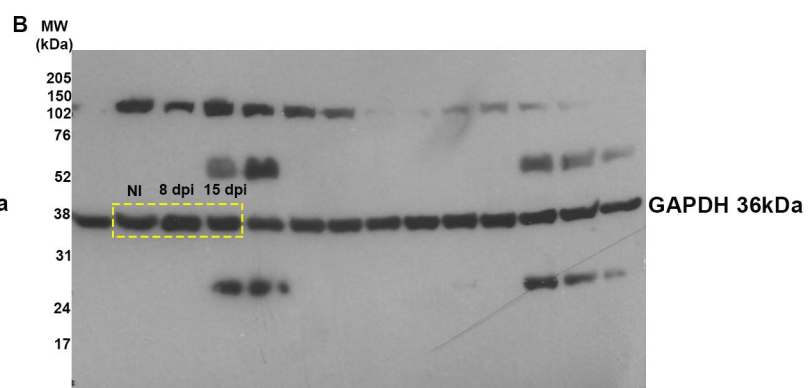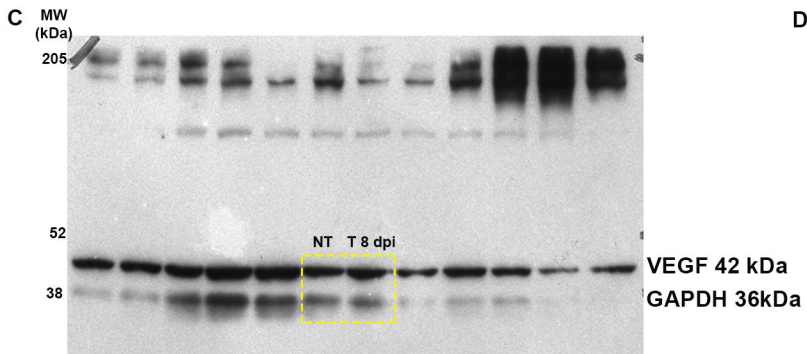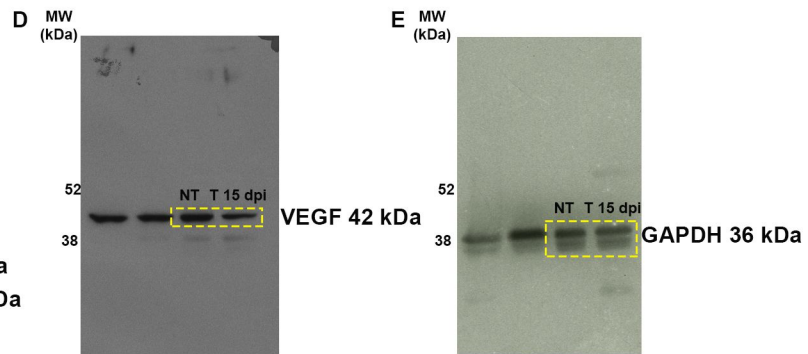

Supplement: Supplementary file 1 [file biology-12-01414-s001.zip › biology-2603819-supplementary.pdf]
